# Integrated Analysis of Angiogenesis Related lncRNA-miRNA-mRNA in Patients With Coronary Chronic Total Occlusion Disease

**DOI:** 10.3389/fgene.2022.855549

**Published:** 2022-04-25

**Authors:** Wei Gao, Jianhui Zhang, Runda Wu, Jie Yuan, Junbo Ge

**Affiliations:** Department of Cardiology, Zhongshan Hospital, Fudan University, Shanghai, China

**Keywords:** coronary artery disease, chronic total occlusion, angiogenesis, lncRNA, mRNA, miRNA

## Abstract

**Background:** Coronary chronic total occlusion (CTO) disease is common and its specific characteristic is collateral formation. The Integrated analysis of angiogenesis related lncRNA-miRNA-mRNA network remains unclear and might provide target for future studies.

**Methods:** A total of five coronary artery disease (control group) and five CTO (CTO group) patients were selected for deep RNA and miRNA sequencing. The expression profiles of lncRNAs, mRNAs circRNA and miRNAs were obtained. Gene Ontology (GO) and Kyoto Encyclopedia of Genes and Genomes (KEGG) pathway enrichment analyses were then performed. The expression of a 14q32 miRNA gene cluster, including miRNA-494, miRNA-495 and miRNA-329, were selected to be determined in another larger patient cohort. Analysis of the lncRNA-miRNA495-mRNA network was constructed to find potential targets for future studies.

**Results:** A total of 871 lncRNAs, 1,080 mRNAs, 138 circRNAs and 56 miRNAs were determined as differentially expressed (DE) in CTO patients compared with control patients. GO and KEGG analyses revealed that the top terms included MAPK signaling pathway, HIF-1 signaling pathway, EGFR tyrosine kinase inhibitor resistance, embryonic organ development, wound healing, MAPK signaling pathway and JAK-STAT signaling pathway, which are related to angiogenesis. The expression of miRNA-494, miRNA-495 and miRNA-329 were all significantly down-regulated in CTO patients and they were confirmed to be down-regulated in another cohort of 68 patients. Then we divided the CTO patients into two groups according to CC grade (poor CC group, CC = 0 or one; good CC group, CC = 2). MiRNA-494, miRNA-495 and miRNA-329 were found to be down-regulated in good CC group compared with poor CC group. Analysis of the lncRNA-miRNA495-mRNA network showed 3 DE lncRNA sponges (NONHSAG008675, NONHSAG020957 and NONHSAG010989), 4 DE lncRNA targets (NONHSAT079547.2, NONHSAT081776.2, NONHSAT148555.1 and NONHSAT150928.1) and 2 DE mRNA targets (RAD54L2 and ZC3H4) of miRNA495.

**Conclusion:** This study revealed that the lncRNA-miRNA-mRNA network might play a critical role in angiogenesis in CTO patients.

## Introduction

Cardiovascular disease is still the leading cause of death worldwide. The prevalence of chronic total occlusions (CTO) among patients with known coronary artery disease (CAD) can be as high as 30–50% (Srinivas et al., 2002; Christofferson et al., 2005). CTO disease is defined as a complete vessel occlusion of native coronary artery and the estimated occlusion duration is more than 3 months. If the relief of angina symptoms and/or an ischemia reduction in the CTO territory can be expected, current guidelines recommended that revascularization can be considered (Windecker et al., 20142014). Thus, new methods or biomarkers screening potential candidates for revascularization are of interest of both clinical and basic science researchers.

One of the specific characteristics of CTO is collateral formation, which can be seen in a majority of these patients (Werner, 2014). Although well-developed collaterals are not enough to provide sufficient blood supply (Sachdeva et al., 2014), they have been demonstrated to be related with improved ventricular function (Werner et al., 2005; Choi et al., 2013). According to the collateral size and filling, collateral vessels can be classified by collateral connection (CC) grade (Meisel et al., 2013). CC grade was defined as follows: CC 0, no continuous connection between donor and recipient artery; CC 1, continuous, threadlike connection (diameter ≤0.3 mm); CC 2, continuous, small, side-branch-like size of the collateral throughout its course (diameter ≥0.4 mm). The major two processes of collateral growth are arteriogenesis and angiogenesis (Zimarino et al., 2014). The underlying mechanisms were complex, including shear stress and molecular and cellular response to hypoxia.

MicroRNAs (miRNAs) are small non-coding RNA molecules and are well investigated in last decades. They have been demonstrated to play an important role in angiogenesis. The roles of miRNAs have been deeply studied in stable CAD (Economou et al., 2015) and myocardial infarction (Chistiakov et al., 2016). However, expression profile and underline mechanisms of miRNAs in CTO patients have not been thoroughly investigated. Recently, long noncoding RNAs (lncRNAs) (Zhao et al., 2020) and circular RNAs (circRNAs) (Liu et al., 2020) have been suggested to be associated with angiogenesis. A complicated network of lncRNAs, circRNAs and miRNAs has been proposed to regulate mRNA function. Therefore, it is necessary to investigate lncRNA-miRNA-mRNA regulatory networks to comprehensively understand their roles in angiogenesis in CTO patients.

In this study, we determined miRNA, circRNAs, mRNAs and lncRNAs expression profiles in CTO patients with deep RNA sequencing (RNA-seq). Gene Ontology (GO) and Kyoto Encyclopedia of Genes and Genomes (KEGG) analyses were performed to explore the potential regulatory functions of differentially expressed (DE) profiles. A network analysis between lncRNA-miRNA-mRNA was also conducted. Then we focused on a 14q32 miRNA gene cluster, including miRNA-494, miRNA-495 and miRNA-329, which were confirmed to be down-regulated in another larger sample of CTO patients.

## Methods

### Patients

A total of five CAD (control group) and five CTO (CTO group) patients were selected for deep RNA and miRNA sequencing. They were all male, aged from 45 to 65 years old, with no left ventricular dysfunction/myocardial infarction history/tumor history, normal liver and renal function. All of the 10 samples were analyzed for lncRNA, mRNA and circRNA sequencing. For miRNA sequencing, five CAD and four CTO samples (the other one was abandoned because of RNA degradation) were analyzed. We selected another cohort of 68 patients to verify expression of miRNAs, including 22 CAD and 46 CTO patients. The CAD patients had a 50–90% stenosis of at least one main vessel. The exclusive criteria were: symptomatic peripheral arterial disease, recent ST-segment elevation myocardial infarction, decompensated heart failure, any concomitant inflammation or infectious diseases, neoplastic diseases, and severe liver and kidney dysfunction. We collected all the patients’ venous blood sample upon hospitalization (within 24 h). The blood sample was centrifuged at 1,500 g for 10 min to precipitate blood cells and plasma was then frozen at −80°C until use.

This study was approved by the medical ethics committee of Zhongshan Hospital. Informed consent was obtained from all patients. All procedures performed in studies involving human participants were in accordance with the ethical standards of the institutional and/or national research committee and with the Helsinki declaration and its later amendments.

### RNA Extraction

RNAs were isolated from the patients’ plasma using TRIzol reagent (Life Technologies). RNA concentrations were measured with a NanoDrop ND-2000 instrument (Thermo Fisher Scientific) and miRNA concentrations were measured with a NanoDrop ND-1000 instru-ment (Thermo Fisher Scientific). Then the high-tthroughput sequencing was performed by WuXi NextCODE company (Shanghai, China) (Cui et al., 2020; Li et al., 2020).

### MiRNA Sequencing

NEB Next Multiplex Small RNA Library Prep Set for Illumina (New England Biolabs) was performed to construct miRNA sequencing libraries. The quality of the libraries was evaluated by a Bioanalyser 2100 system (Agilent Technologies). A HiSeq 4000 sequencing system (Illumina) was used for sequencing for 50 cycles according to the manufacturer’s instructions. Cutadapt software (v1.9.3) was used to trim the adaptor sequences from the sequencing data. Trimmed readswere aligned tohuman reference genome (hg19) using Bowtie. Mapped reads were used to identify known miRNAs applying miRBasev21. MiRDeep2 weas used to predict novel miRNAs.

### RNA Sequencing

TruSeq Stranded Total RNA Library Prep Kit (Illumina) was used to construct RNA sequencing libraries. The quality was evaluated by a Bioanalyser 2100 system (Agilent Technologies). Then, single-stranded DNA molecules were clustered and sequenced for 150 cycles on an Illumina HiSeq 2500 sequencing system (Illumina). Paired-end reads were acquired from the Illumina HiSeq sequencer, and the quality control was performed with Q30. Cutadapt software (v1.9.3) was used for removal of 3’ adaptor-trimming and low-quality reads. Alignment of the high-quality clean reads to the human reference genome (hg19) was performed by hisat2 (v2.0.4). The transcriptome was assembled using Cufflinks. Cuffcompare was used to identify known lncRNAs in NONCODE V5.0 and novel lncRNAs. Acfs was used to identify circRNAs. Then we used circBase to separate known circRNAs and novel circRNAs.

### Differentially Expressed Analysis

EdgeR was used to identify differential expressed miNRA, mRNAs, lncRNAs and circRNAs (Benjamini Hochber method corrected *p*-value <0.05, Fold change >2) for the compare between control and CTO group.

### Functional Enrichment Analysis

We used “ClusterProfiler” package in R software to perform functional enrichment analysis, and GO biological processes and KEGG pathways at the significant level (q-value < 0.01) were employed.

### Construction of the lncRNA-miRNA-mRNA Network

The lncRNA-miRNA interactions were predicted by NPinter v4.0 and LncBase v3.0. We merged interactions with strong and weak evidence. We required each lncRNA-miRNA interactions shared with miRNA more than two times. And the lncRNA-miRNA interactions should be both significantly up/down-regulated expressed with adjusted *p*-value < 0.01. The targets of differentially expressed miRNA were predicted by miRTarBase. Also, mRNA-miRNA pairs should be both significantly expressed with adjusted *p*-value < 0.01. The network was plotted by Cytoscape.

### Reverse Transcription-PCR

Total RNAs were extracted from plasma with TRIzol reagent according to manufacturer’s protocol (Invitrogen; Thermo Fisher Scientific, Inc.). MicroRNA qRT-PCR syb kit (Taqman) was used for generating cDNA from microRNA and qPCR detection. We used ABI 7500 real-time PCR system to perform real-time PCR. The qPCR conditions were as follows: 95°C for 15 min, 30 cycles of 95°C for 5 s, and 60°C for 30 s. The relative expression of the miRNAs was normalized to that of U6 by using the 2-ΔΔCq cycle threshold method. We used Student t test to determine statistical significance between the groups. All statistical analyses were performed by SPSS 17.0 (SSPS Inc., Chicago, IL, United States). A *p* < 0.05 was considered significant.

## Results

### Characteristics of Patients

The patients’ baseline characteristics were shown in Table 1. Briefly, they were all male, aged from 45 to 65 years old, with no renal dysfunction and heart failure. The CAD patients were all presented with left anterior descending (LAD) stenosis of 70–90%. As for CTO vessel in CTO group, all the occluded vessels are LADs. All of the five CTO patients for RNA sequencing had good collaterals with CC grade of 2.

**TABLE 1:** Baseline characteristics of included patients.

	CON-1	CON-2	CON-3	C0N-4	CON-5	CTO-1	CTO-2	CTO-3	CTO-4	CTO-5
Age	64	64	65	58	48	62	58	65	64	63
Gender	Male	Male	Male	Male	Male	Male	Male	Male	Male	Male
Hypertension	0	1	1	0	0	0	0	1	0	0
Diabetes	0	0	0	0	0	0	0	0	0	0
Smoking	0	1	0	0	0	0	1	0	0	1
Cr (umol/L)	100	70	95	98	80	73	91	78	65	70
LDL-C (mmol/L)	2.48	1.93	4.9	1.46	1.59	1.05	1.48	1.76	1.17	2.2
TNT (ng/ml)	0.009	0.008	0.004	0.005	0.014	0.007	0.012	0.012	0.022	0.019
NT-proBNP (pg/ml)	178	52.9	140	53.9	135	307	210	30.9	95.2	85
LVEF (%)	69	67	63	64	70	64	70	66	65	73

Cr, creatinine; LDL-C, low density lipoprotein cholesterol; TNT, troponin T; NT-proBNP, N-terminal pro brain natriuretic peptide; LVEF, left ventricular ejection fraction.

### Overview of lncRNA, mRNA, circRNA and miRNA Expression in CTO Patients

**FIGURE 1:**
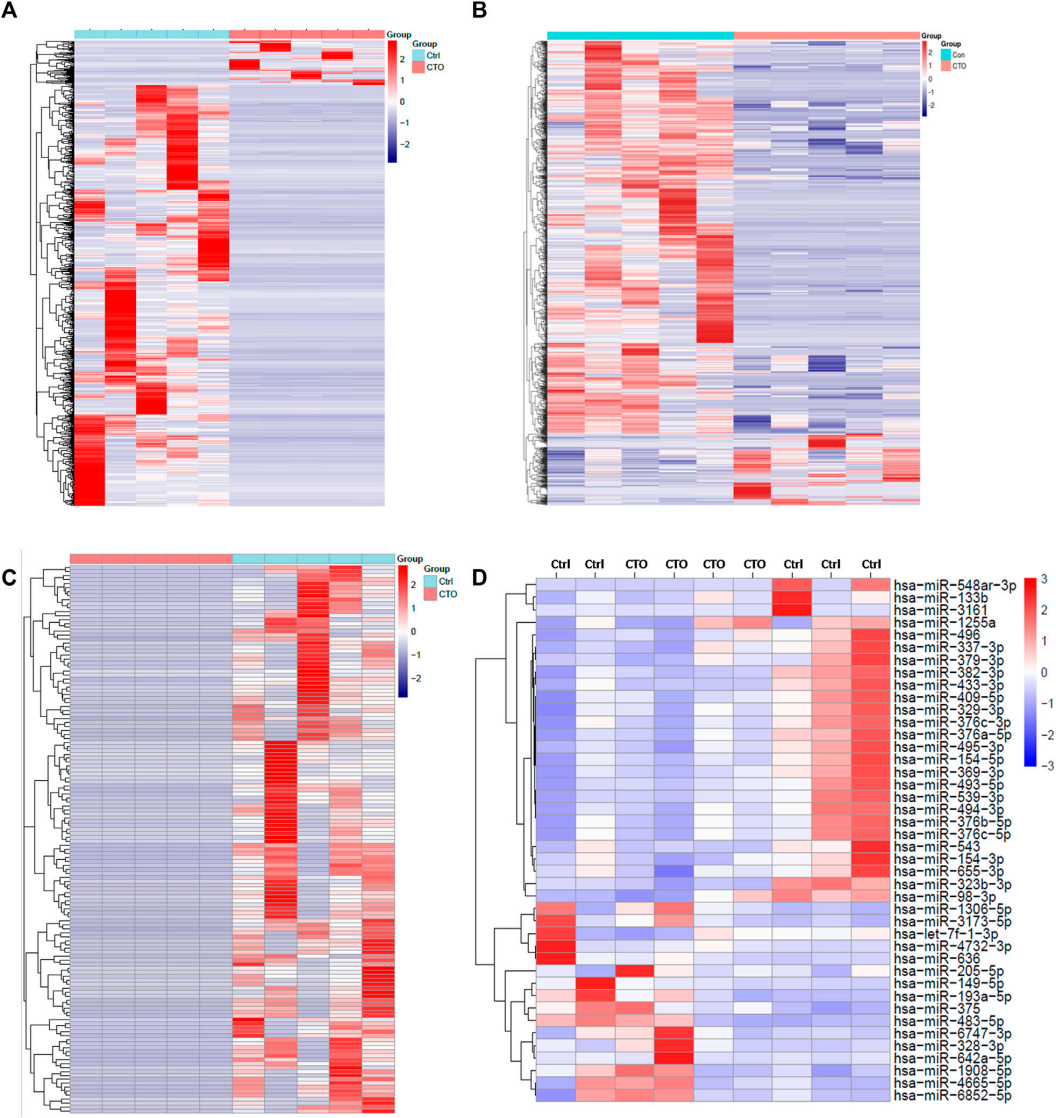
Expression profile of lncRNAs, mRNAs, circRNAs and miRNAs. Hierarchical clustering of DE lncRNAs **(A)**, mRNAs **(B)**, circRNA **(C)** and miRNAs **(D)** were showed in heatmaps.

The expression heatmaps of lncRNA, mRNA, circRNA and miRNA were shown in Figure 1. Compared with control samples, the CTO samples had 871 significantly and DE lncRNA transcripts, of which 83 were up-regulated and 788 were down-regulated. For mRNA, there were 1,080 significantly and DE mRNA transcripts, of which 166 transcripts were up-regulated and 914 were down-regulated. For circRNA, there were 138 significantly and DE circRNA transcripts and all of them were down-regulated. Finally, we detected 56 DE miRNAs between the two groups, of which 24 were up-regulated and 32 were down-regulated. The top significantly up-regulated and down-regulated lncRNA, mRNA, circRNA and miRNA were summarized in Tables 2–5.

**TABLE 2:** Top 10 significantly upregulated and downregulated lncRNA between groups.

lncRNA_id	Locus	*p* value	Q Value	Status
NONHSAT150928.1	1:629661-630563	0.001157	0.884375	Up-regulated
MSTRG.90871.3	4:7130626-7132025	0.010302	0.884375	Up-regulated
MSTRG.60272.4	19:35297866-35298779	0.032029	0.884375	Up-regulated
MSTRG.127463.1	8:142936802-142937536	0.032602	0.884375	Up-regulated
MSTRG.90672.6	4:3678318-3678885	0.001302	0.884375	Up-regulated
MSTRG.31433.9	12:125409520-125411209	0.042556	0.884375	Up-regulated
MSTRG.31433.17	12:125409529-125411210	0.013733	0.884375	Up-regulated
NONHSAT188821.1	20:46207026-46212517	0.021585	0.884375	Up-regulated
MSTRG.90672.3	4:3678038-3678904	0.006796	0.884375	Up-regulated
NONHSAT160436.1	11:65854817-65860166	0.041821	0.884375	Up-regulated
NONHSAT166700.1	13:44434118-44436839	0.020271	0.884375	Down-regulated
ENST00000451706	10:120763401-120793525	0.000181	0.665789	Down-regulated
NONHSAT064188.2	19:32206061-32216482	0.002338	0.884375	Down-regulated
ENST00000605901	6:2988648-2991173	0.000147	0.665789	Down-regulated
NONHSAT152519.1	1:169588854-169593726	1.37E-05	0.338588	Down-regulated
NONHSAT200055.1	4:73996609-73998667	0.009095	0.884375	Down-regulated
NONHSAT222650.1	X:41085467-41136929	2.63E-05	0.338588	Down-regulated
NONHSAT159621.1	11:108008936-108054788	6.53E-05	0.560167	Down-regulated
NONHSAT206983.1	6:26204366-26204807	0.006172	0.884375	Down-regulated
NONHSAT207060.1	6:29928300-29945868	0.014236	0.884375	Down-regulated

**TABLE 3:** Top 10 significantly upregulated and downregulated mRNA between groups.

Gene Id	Gene name	*p* value	Q Value	Status
ENSG00000198886	MT-ND4	5.42E-05	0.078103	Up-regulated
ENSG00000198840	MT-ND3	0.000674	0.256288	Up-regulated
ENSG00000212907	MT-ND4L	0.002148	0.424011	Up-regulated
ENSG00000198899	MT-ATP6	0.000778	0.26115	Up-regulated
ENSG00000215326	GPX1P2	0.019704	0.875439	Up-regulated
ENSG00000264484	RN7SL697P	0.032837	0.975434	Up-regulated
ENSG00000273058	RP11-385F5.5	0.036314	0.975434	Up-regulated
ENSG00000261915	RP11-542C16.2	1.39E-06	0.007558	Up-regulated
ENSG00000223350	IGLV9-49	0.028725	0.957914	Up-regulated
ENSG00000264745	TTC39C-AS1	0.013592	0.804002	Up-regulated
ENSG00000211935	IGHV1-3	1.64E-05	0.044486	Down-regulated
ENSG00000086506	HBQ1	3.92E-05	0.078103	Down-regulated
ENSG00000164047	CAMP	0.000125	0.105849	Down-regulated
ENSG00000237419	RP11-885N19.6	0.00178	0.389996	Down-regulated
ENSG00000235499	AC073046.25	0.020497	0.875439	Down-regulated
ENSG00000262664	OVCA2	1.72E-05	0.044486	Down-regulated
ENSG00000164821	DEFA4	0.005826	0.619616	Down-regulated
ENSG00000250318	CTA-963H5.5	0.007062	0.6687	Down-regulated
ENSG00000258659	TRIM34	2.19E-10	5.66E-06	Down-regulated
ENSG00000269946	RP11-2B6.3	0.018714	0.871651	Down-regulated

**TABLE 4:** Top 10 significantly downregulated circRNA between groups.

circRNA_id	Known_circRNA	Gene_id	*p* value	Q Value	Status
circRNA.2642	—	ENSG00000102580	0.004947	0.920758	Down-regulated
circRNA.10222	hsa_circ_0067900	ENSG00000173889	0.001423	0.920758	Down-regulated
circRNA.9697	hsa_circ_0008768	ENSG00000151693	0.001	0.920758	Down-regulated
circRNA.9677	hsa_circ_0000972	ENSG00000143797	0.001248	0.920758	Down-regulated
circRNA.15109	hsa_circ_0004425	ENSG00000251349	0.034481	0.920758	Down-regulated
circRNA.8956	hsa_circ_0058493	ENSG00000144468	0.007753	0.920758	Down-regulated
circRNA.15168	hsa_circ_0088051	ENSG00000106868	0.013037	0.920758	Down-regulated
circRNA.8516	hsa_circ_0004491	ENSG00000115947	0.00129	0.920758	Down-regulated
circRNA.10871	hsa_circ_0001432	ENSG00000109323	0.009642	0.920758	Down-regulated
circRNA.11611	—	ENSG00000132405	0.035263	0.920758	Down-regulated

**TABLE 5:** Top 10 significantly upregulated and 15 downregulated miRNA between groups.

miRNA	logFC	*p* value	Q Value	Status
hsa-miR-642a-5p	3.749608	4.86E-06	0.000632	Up-regulated
hsa-miR-6747-3p	2.719619	0.002109	0.033775	Up-regulated
hsa-miR-1306-5p	1.909746	1.82E-07	0.000142	Up-regulated
hsa-miR-375	1.816148	1.01E-05	0.000779	Up-regulated
hsa-miR-1908-5p	1.727125	5.87E-06	0.000654	Up-regulated
hsa-miR-636	1.723784	0.003794	0.04851	Up-regulated
hsa-miR-483-5p	1.661883	1.57E-06	0.000409	Up-regulated
hsa-miR-193a-5p	1.653385	1.07E-06	0.000409	Up-regulated
hsa-miR-328-3p	1.593131	6.88E-06	0.000671	Up-regulated
hsa-miR-4732-3p	1.548014	0.001097	0.025166	Up-regulated
hsa-miR-3161	−8.26953	0.001284	0.025834	Down-regulated
hsa-miR-1255a	−4.10217	7.99E-05	0.003463	Down-regulated
hsa-miR-548ar-3p	−3.7005	0.000162	0.005738	Down-regulated
hsa-miR-133b	−2.45368	0.001637	0.030029	Down-regulated
hsa-miR-433-3p	−2.07696	1.10E-05	0.000779	Down-regulated
hsa-miR-154-3p	−2.04281	0.001193	0.025834	Down-regulated
hsa-miR-337-3p	−2.03151	4.63E-06	0.000632	Down-regulated
hsa-miR-376b-5p	−1.98449	3.93E-05	0.002192	Down-regulated
hsa-miR-376c-5p	−1.98246	3.91E-05	0.002192	Down-regulated
hsa-miR-323b-3p	−1.91492	3.10E-06	0.000605	Down-regulated
hsa-miR-494-3p	−1.87927	3.56E-05	0.002192	Down-regulated
hsa-miR-495-3p	−1.85345	4.97E-05	0.002583	Down-regulated
hsa-miR-655-3p	−1.81417	0.003071	0.042029	Down-regulated
hsa-miR-369-3p	−1.81245	8.26E-06	0.000716	Down-regulated
hsa-miR-379-3p	0.301606297	0.000667	0.019259	Down-regulated

### Functional Annotation: Gene Ontology and Kyoto Encyclopedia of Genes and Genomes

GO and KEGG analyses were conducted on the differential expressed lncRNA, mRNA, circRNA and miRNA. For lncRNA, the top 20 GO-BP terms and top 30 KEGG terms were shown in Supplementary Figures S1, S2. For mRNA, the top 10 GO-BP terms was shown in Supplementary Figure S3 and no enriched KEGG pathways were found. For circRNA, the top 20 GO-BP terms and top 30 KEGG terms were shown in Supplementary Figures S4, S5. For miRNA, the top GO-BP terms and top KEGG terms were shown in Supplementary Figures S6, S7. We noticed that the top terms included AMPK signaling pathway, HIF-1 signaling pathway, EGFR tyrosine kinase inhibitor resistance, embryonic organ development, wound healing, MAPK signaling pathway and JAK-STAT signaling pathway, which are related to angiogenesis.

### Construction of the lncRNA-miRNA-mRNA Network

**FIGURE 2:**
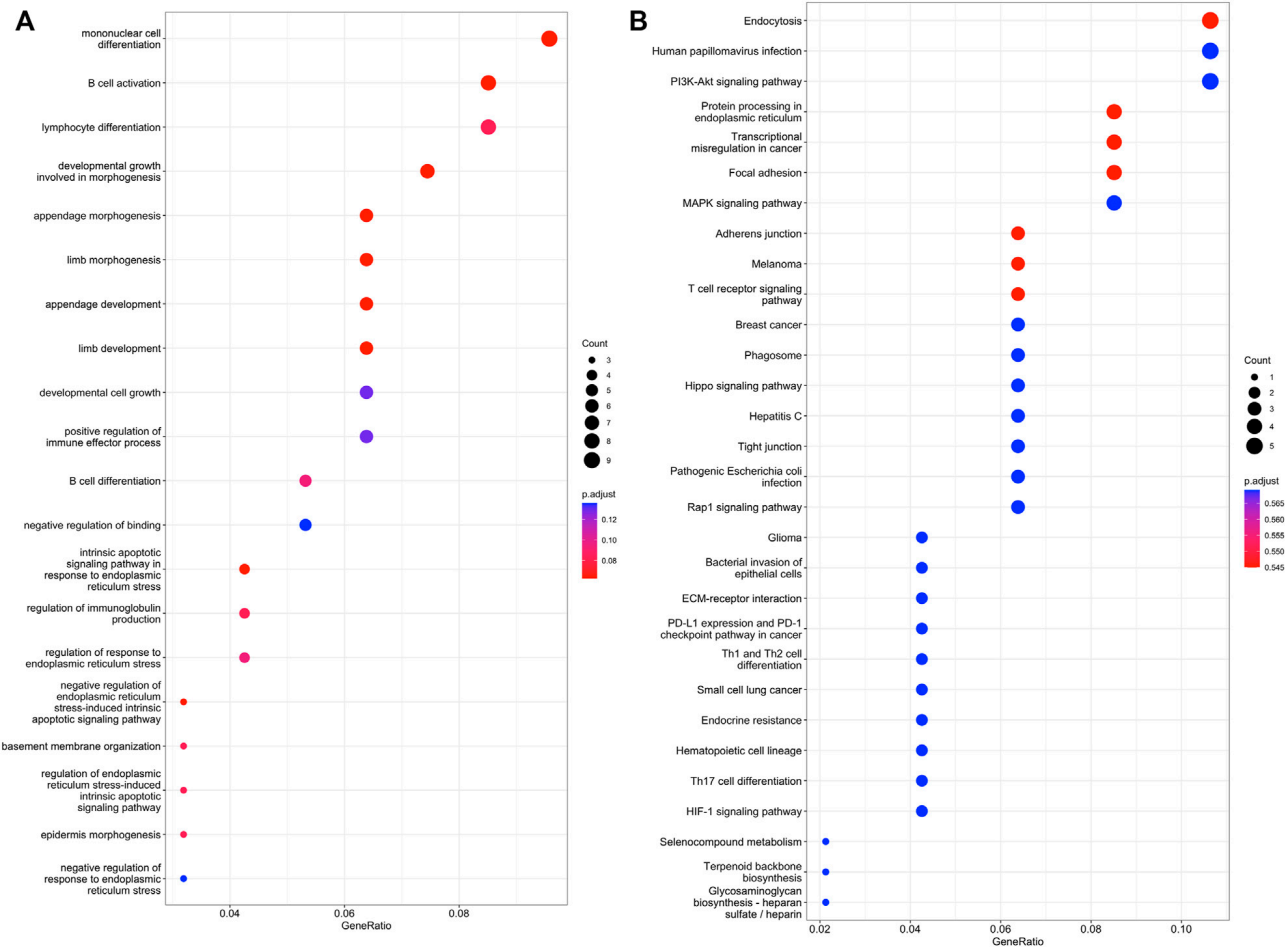
GO and KEGG pathway analysis of DE lncRNA-miRNAs and miRNA-mRNA networks. **(A)**, the top 20 GO terms. **(B)**, the top 30 KEGG terms.

RNA transcripts can effectively interact with one another based on the ceRNA hypothesis. LncRNAs can absorb miRNAs by binding to miRNAs and subsequently exhibiting a miRNA sponge function. Prediction results from bioinformatic analyses indicated that 32 lncRNAs were targeted by 13 miRNAs; nine lncRNAs potentially acted as decoys for five miRNAs. Based on the lncRNA-miRNA and miRNA-mRNA interaction pairs, a lncRNA-miRNA-mRNA network was constructed (Supplementary Figures S8). The network consisted of 46 lncRNA-miRNA relationship pairs and 199 miRNA-mRNA relationship pairs. The top GO-BP and KEGG terms of lncRNA-miRNA-mRNA network analysis were shown in Figure 2. Some angiogenesis related pathways were also observed, such as MAPK and HIF-1 signaling pathway.

### MiRNA 494, 495 and 329 Expressions in Control and CTO Patients

**FIGURE 3:**
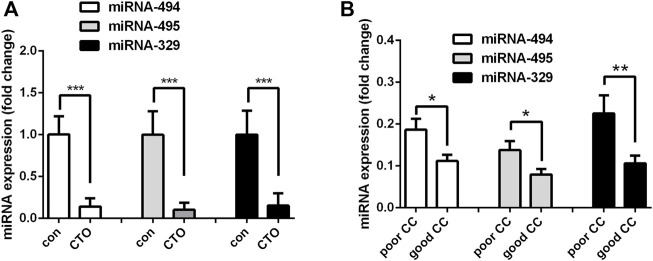
Expression of miR-494, miR-495 and miR-329 in another patient cohort. **(A)**, Expressions of these miRNAs in patients with coronary artery disease (control group, *N* = 22) and chronic total occlusions (CTO group, *N* = 46). **(B)**, Patients in CTO group were divided into two subgroups according to CC grade. Expression levels of miRNA-329, miRNA-494 and miRNA-495 in poor CC group (*N* = 17, CC = 0 or 1) and good CC group (*N* = 27, CC = 2). *p < 0.05, **p < 0.01, ***p < 0.001.

After analyzing the differentially expressed miRNAs, we noticed that miRNA-494, miRNA-495 and miRNA-329 belong to the 14q32 miRNA gene cluster, which were all significantly down-regulated in CTO patients. The expression of these three miRNAs were then determined in another cohort of 68 male patients, including 22 CAD patients and 46 CTO patients. We confirmed that miRNA-494, miRNA-495 and miRNA-329 were indeed down-regulated in CTO patients (Figure 3A). Then we divided the CTO patients into two groups according to CC grade (poor CC group, CC = 0 or one; good CC group, CC = 2). MiRNA-494, miRNA-495 and miRNA-329 were found to be down-regulated in good CC group compared with poor CC group (Figure 3B). These data indicated that the expression of miRNA-494, miRNA-495 and miRNA-329 were associated with angiogenesis in CTO patients.

### Analysis of the lncRNA-miRNA495-mRNA Network

**FIGURE 4:**
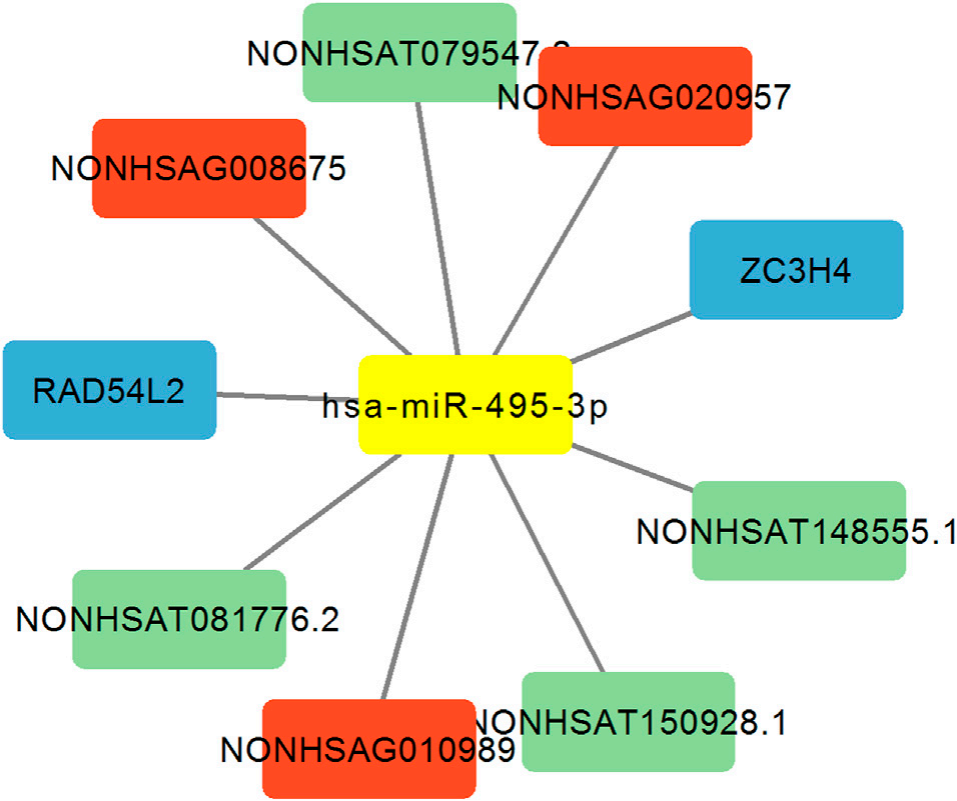
Analysis of the lncRNA-miRNA495-mRNA network. Red indicates lncRNA sponge of miR-495. Blue indicates mRNA target of miR-495. Green indicates lncRNA target of miR-495.

Previous studies found miRNA-329 and miRNA-494 suppresses angiogenesis by targeting CD146 (Wang et al., 2013) and BMPER (Esser et al., 2017), respectively. Whether and how miRNA-495 inhibits angiogenesis of endothelial cells remains unclear. Then we focused on miRNA-495 and constructed the analysis of lncRNA-miRNA495-mRNA network. As shown in Figure 4, the results revealed that miRNA-495 targeted 3 DE lncRNA sponges (NONHSAG008675, NONHSAG020957 and NONHSAG010989), 4 DE lncRNA targets (NONHSAT079547.2, NONHSAT081776.2, NONHSAT148555.1 and NONHSAT150928.1) and 2 DE mRNA targets (RAD54L2 and ZC3H4). These data indicated that miRNA-495, together with the targeted lncRNAs and mRNAs, might play a regulatory role in angiogenesis. Further experiments investigating the underlying mechanisms are of potential value.

## Discussion

The specific characteristic of CTO is collateral circulation, which attracted lots of attention in CTO research. Clinical doctors focused on whether the collateral circulation is sufficient to provide blood supply and can predict clinical prognosis. Basic researchers were interested in how it takes shape and what the impact factors are. In this study, we evaluated the expression profiles of mRANs and non-coding RNAs, including lncRNAs, cirRNAs and miRNAs, in the plasma of CTO patients. We also conducted a network between these RNAs and proposed several potential targets involving into angiogenesis. To our knowledge, this is the first bioinformatics study to comprehensively investigate the RNA expression profile and their network in CTO patients.

lncRNAs are a class of non-coding RNAs that are more than 200 nt in length. In addition to being directly involved in the regulation of gene expression, lncRNA can also function as a competing endogenous RNA, which competes with other RNA transcripts for the same miRNA, resulting in interactions and subsequent regulation. As discussed in a previous review (Zhao et al., 2020), lncRNA-miRNA interaction play a role in angiogenesis through several signaling pathways, including VEGF, Notch, PI3K/AKT, JAK-STAT and ERK (Li et al., 2017; Gao et al., 2018; Wang et al., 2018; Yin et al., 2018; Moradi et al., 2019). In our study, we noticed that a previous reported angiogenesis related lncRNA, TUG1 (ENST0000051907), was differently expressed in CTO patients compared with control patients (not shown in Table). And also, GO and KEGG analyses revealed some angiogenesis related pathways, such as HIF-1 and EGFR tyrosine kinase inhibitor resistance. When focusing on miRNA-495 targeted lncRNAs, we found some new lncRNAs, which are potentially involved into angiogenesis of CTO disease. Future studies focusing on these new lncRNAs are needed.

CircRNAs are a class of novel RNAs that have a special covalent loop structure without a 5′ cap and 3’ tail. The formation mechanism of circRNAs is not well understood. Although circRNAs are less studied compared with lncRNAs or miRNAs, some recent studies have shown that circRNA has a role in the regulation of angiogenesis related diseases, such as atherosclerosis, myocardial hypertrophy and hypertension (Boeckel et al., 2015; Wang et al., 2016; Bao et al., 2018). In our study, the GO and KEGG analyses showed some angiogenesis related pathways, including MAPK and JAK-STAT signaling pathway. Then we used starBase (https://starbase.sysu.edu.cn/) to predict miRNA495-circRNA interactions. Among the top 10 significantly down-regulated circRNAs in Table 4, circRNA.2642 (DNAJC3), circRNA.10222 (PHC3) and circRNA.9677 (MBOAT2) were the targets of miRNA-495. These circRNAs are worthy to be investigated in future studies.

The most deeply studied non-coding RNAs are miRNAs. As discussed in a recent review, miRNAs can be both anti-angiogenic and proangiogenic (Kir et al., 2018). Among the differently expressed miRNAs between control and CTO patients, we focused on a 14q32 miRNA gene cluster, including miRNA-494, miRNA-495 and miRNA-329. In a previous study, Gene-Specific Oligonucleotides (GSO) were used to systemically inhibit these miRNAs in hind limb ischemia models and improved recovery of perfusion was observed (Welten et al., 2014). We found that down-regulation of these three miRNAs were associated with well developed collateral circulation in CTO patients. We further investigated the mechanism of how miRNA-495 regulates angiogenesis in both *in vitro* and *in vivo* study (data not shown). The lncRNA-miRNA495-mRNA network is also of our great interest. In this current study, we showed several lncRNAs and mRNAs targets of miRNA495, which are worthy of more investigation in future studies.

## Conclusion

This is the first bioinformatics study to comprehensively investigate the RNA expression profile and their network in CTO patients. The results showed that lncRNA-miRNA-mRNA network might play a critical role in angiogenesis in CTO patients.

## Data Availability

The datasets presented in this study can be found in online repositories. The names of the repository/repositories and accession number(s) can be found below: https://ncbi.nlm.nih.gov/sra; PRJNA799206.
