# Supplementary figures and images for: Integrated Analysis of Angiogenesis Related lncRNA-miRNA-mRNA in Patients With Coronary Chronic Total Occlusion Disease
Source: Front Genet. 2022 Apr 25;13:855549. doi: 10.3389/fgene.2022.855549 (PMC9081538; doi:10.3389/fgene.2022.855549)

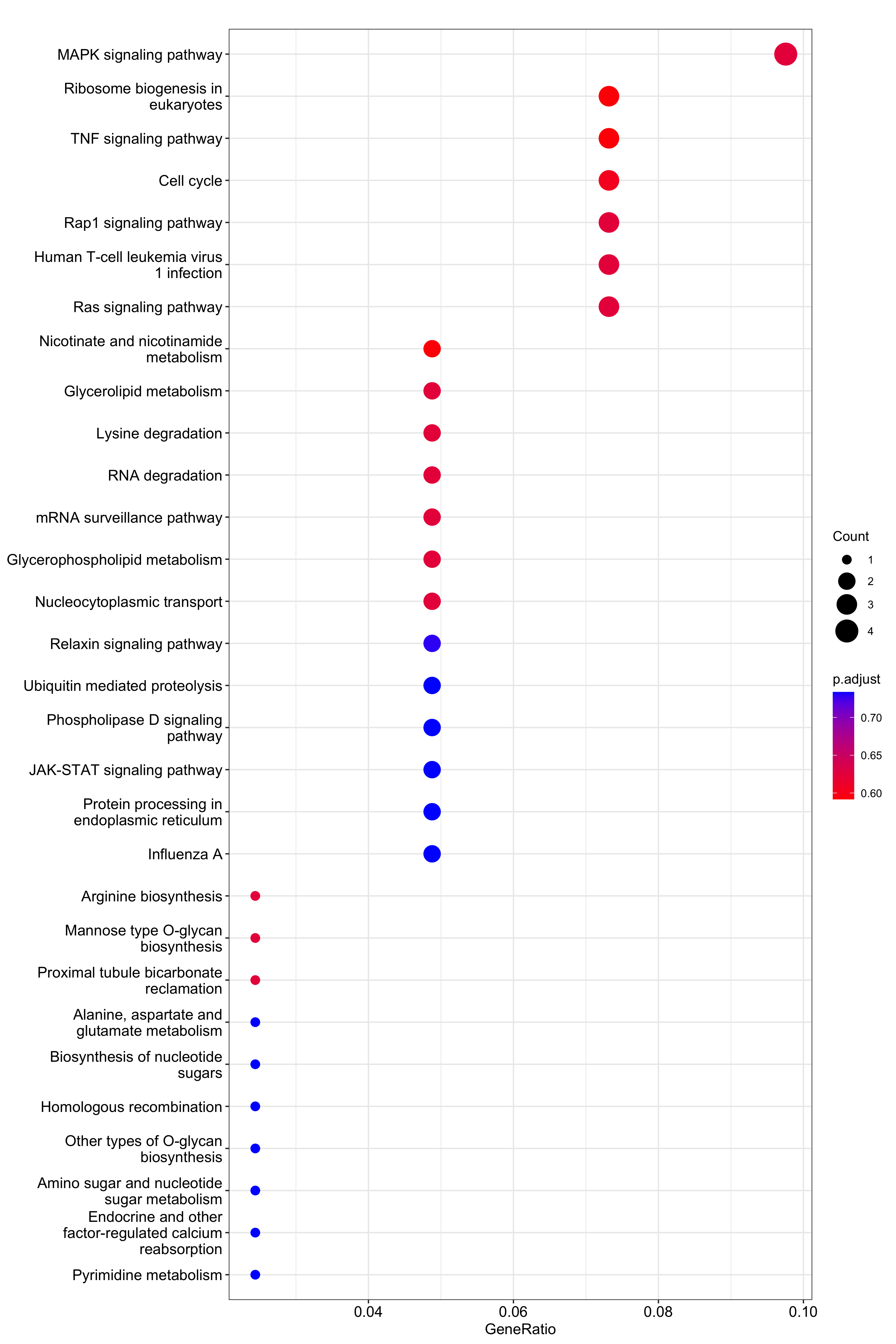

Supplement: Supplementary file 1 [file Image5.PNG]

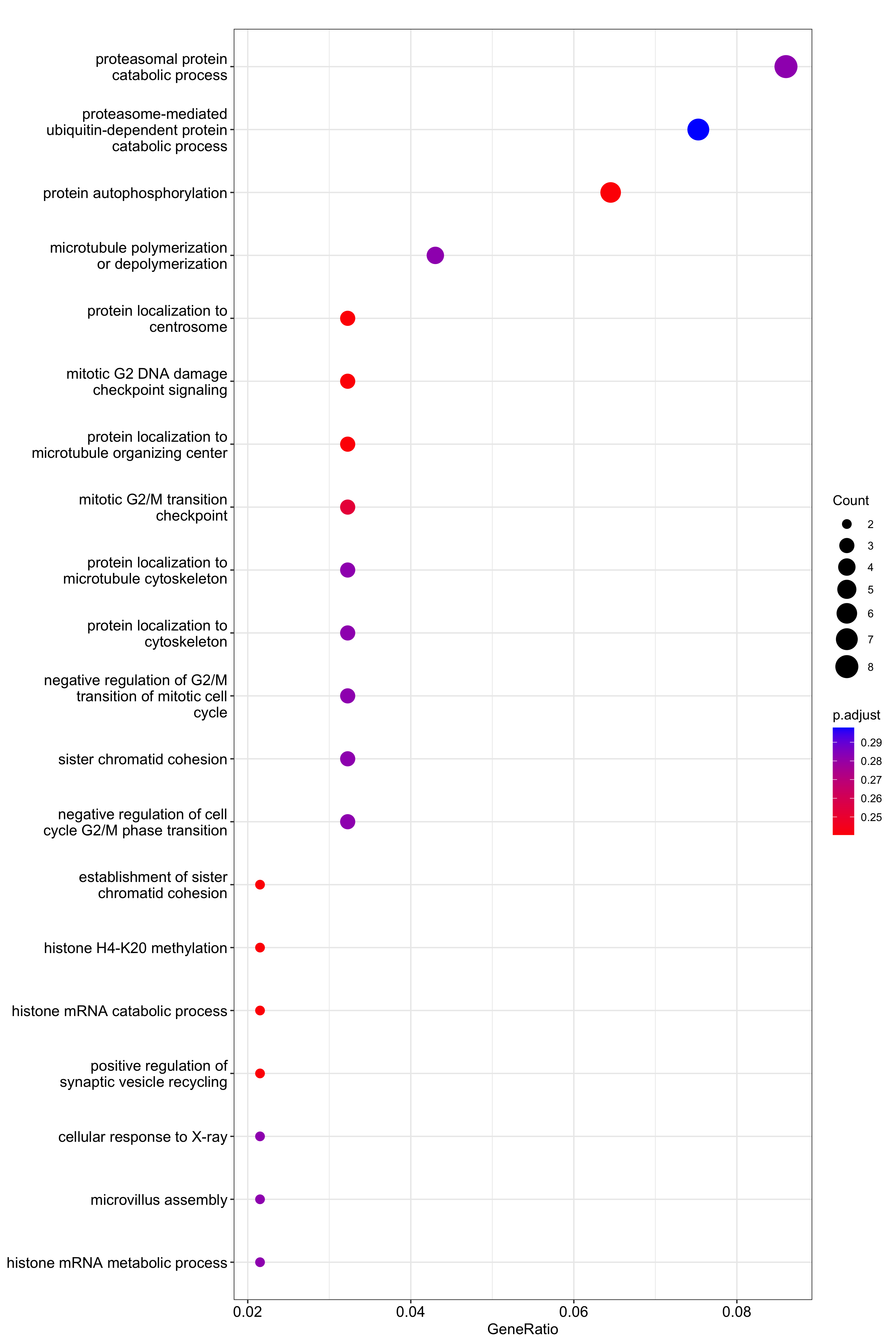

Supplement: Supplementary file 2 [file Image4.PNG]

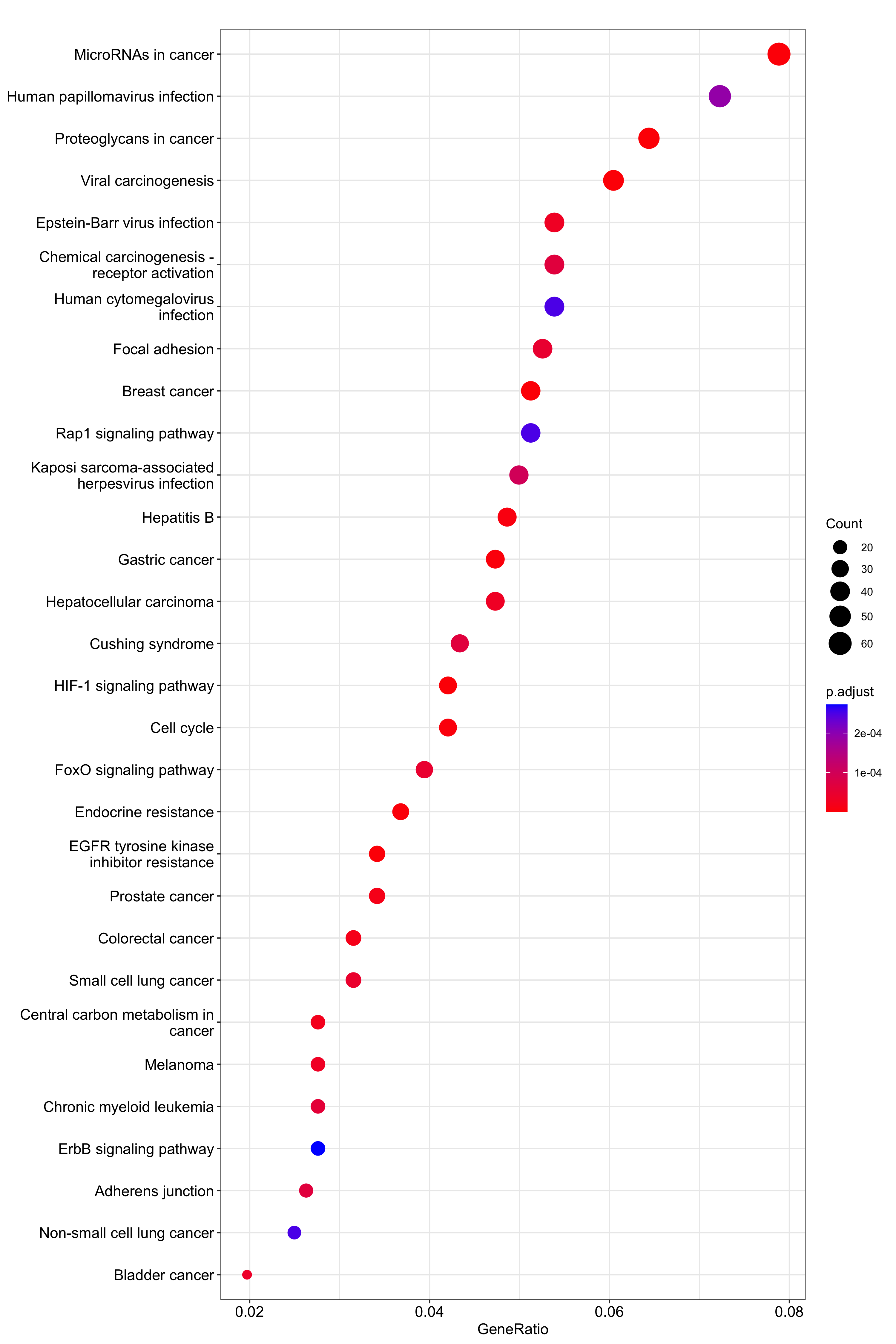

Supplement: Supplementary file 3 [file Image7.PNG]

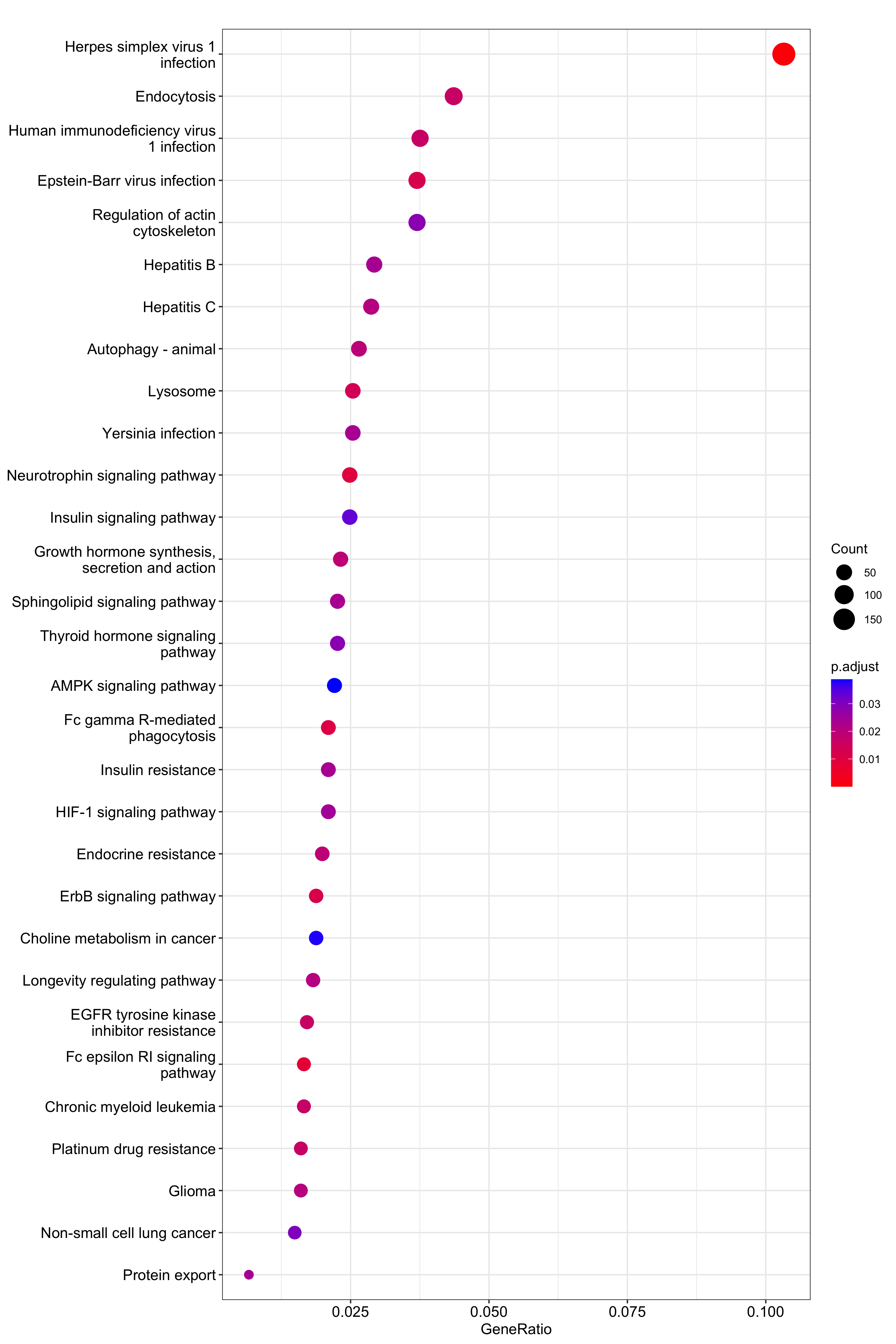

Supplement: Supplementary file 4 [file Image2.PNG]

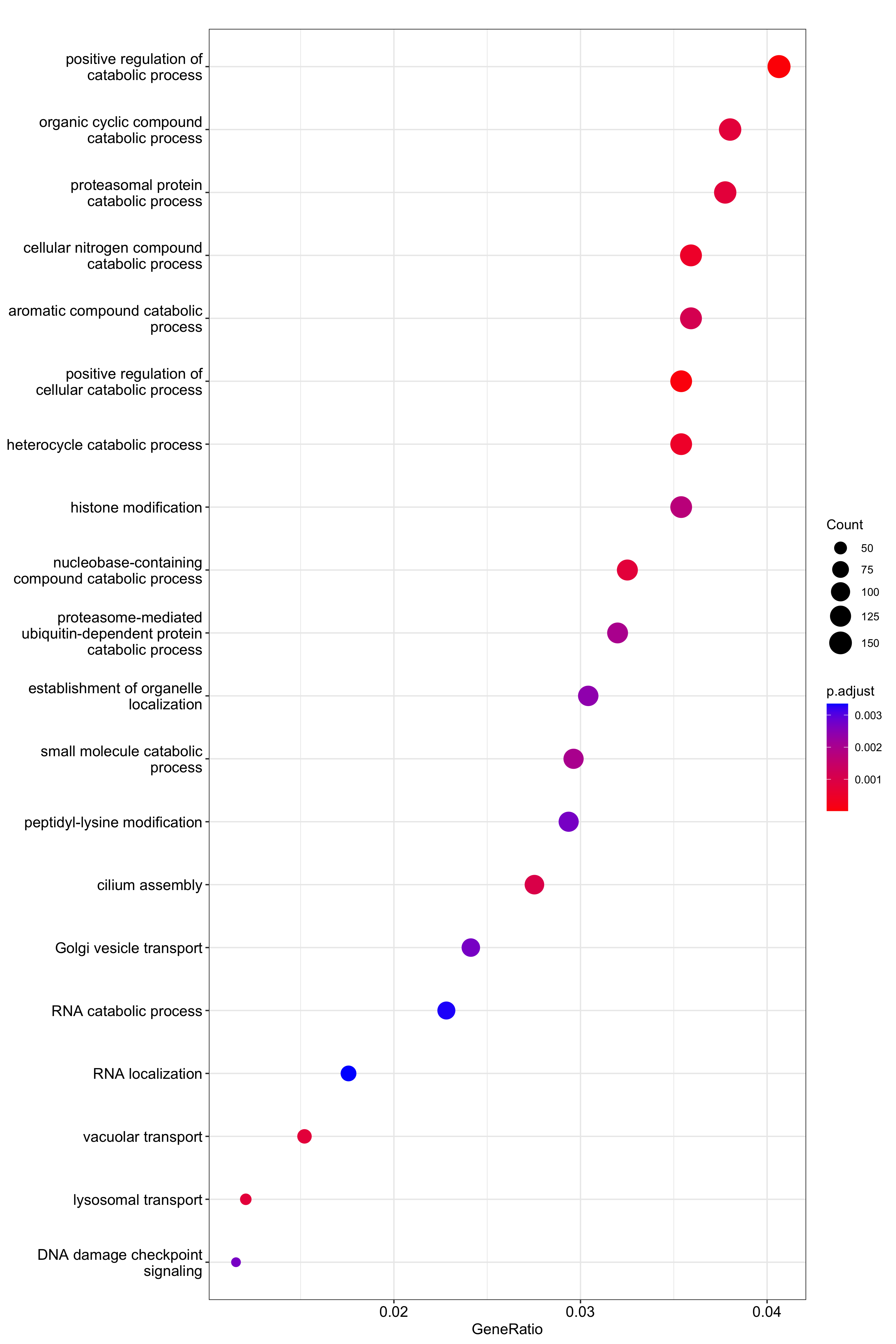

Supplement: Supplementary file 5 [file Image1.PNG]

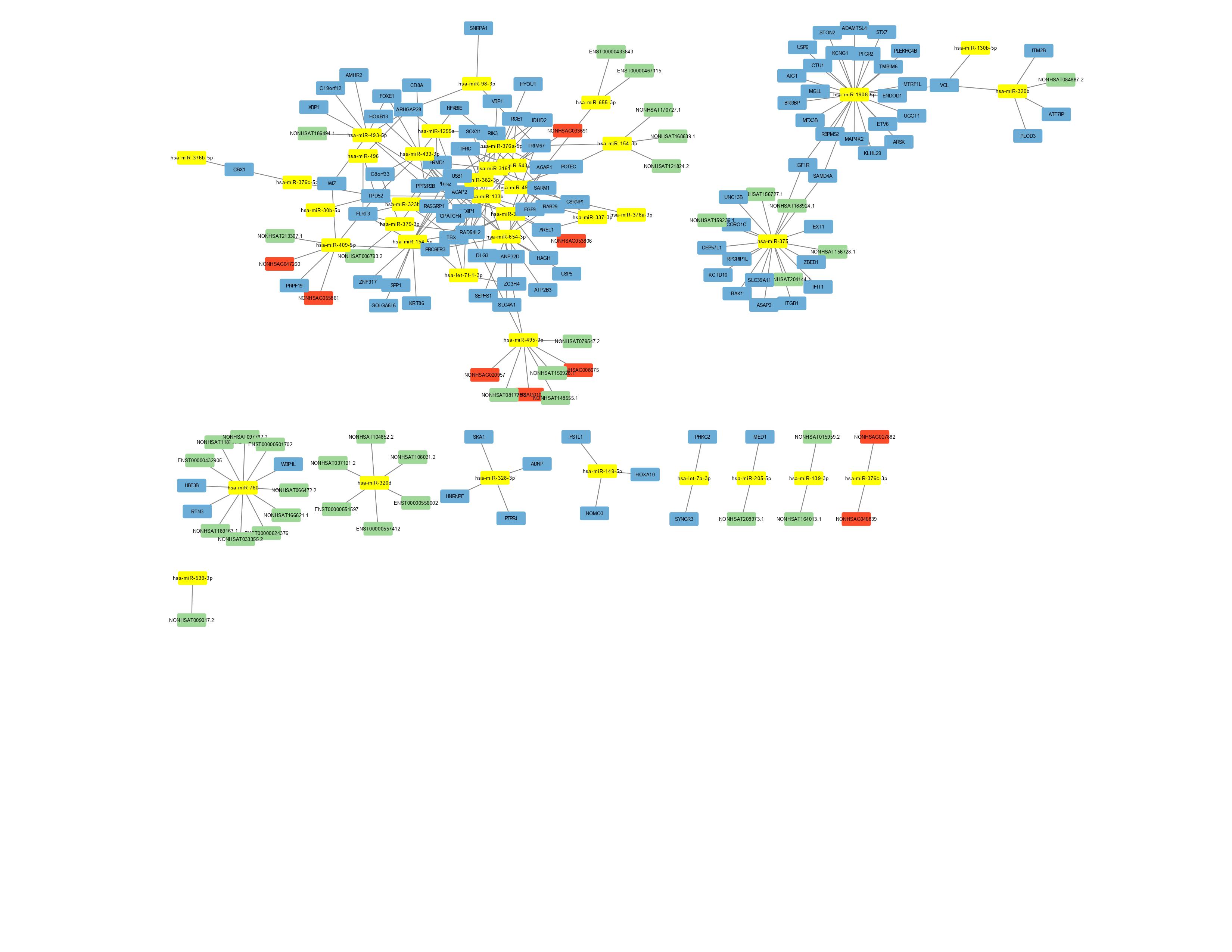

Supplement: Supplementary file 6 [file Image8.JPEG]

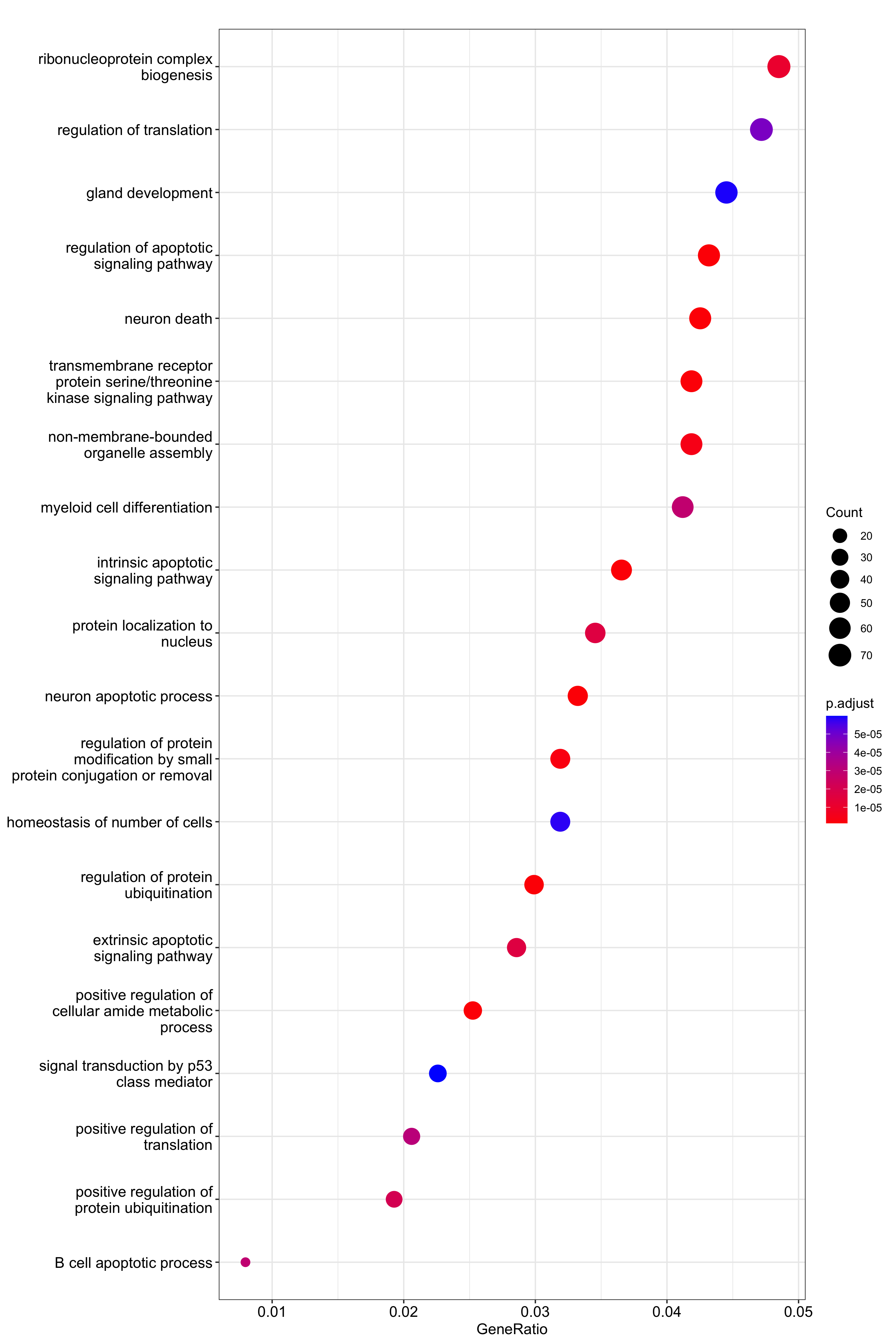

Supplement: Supplementary file 7 [file Image6.PNG]

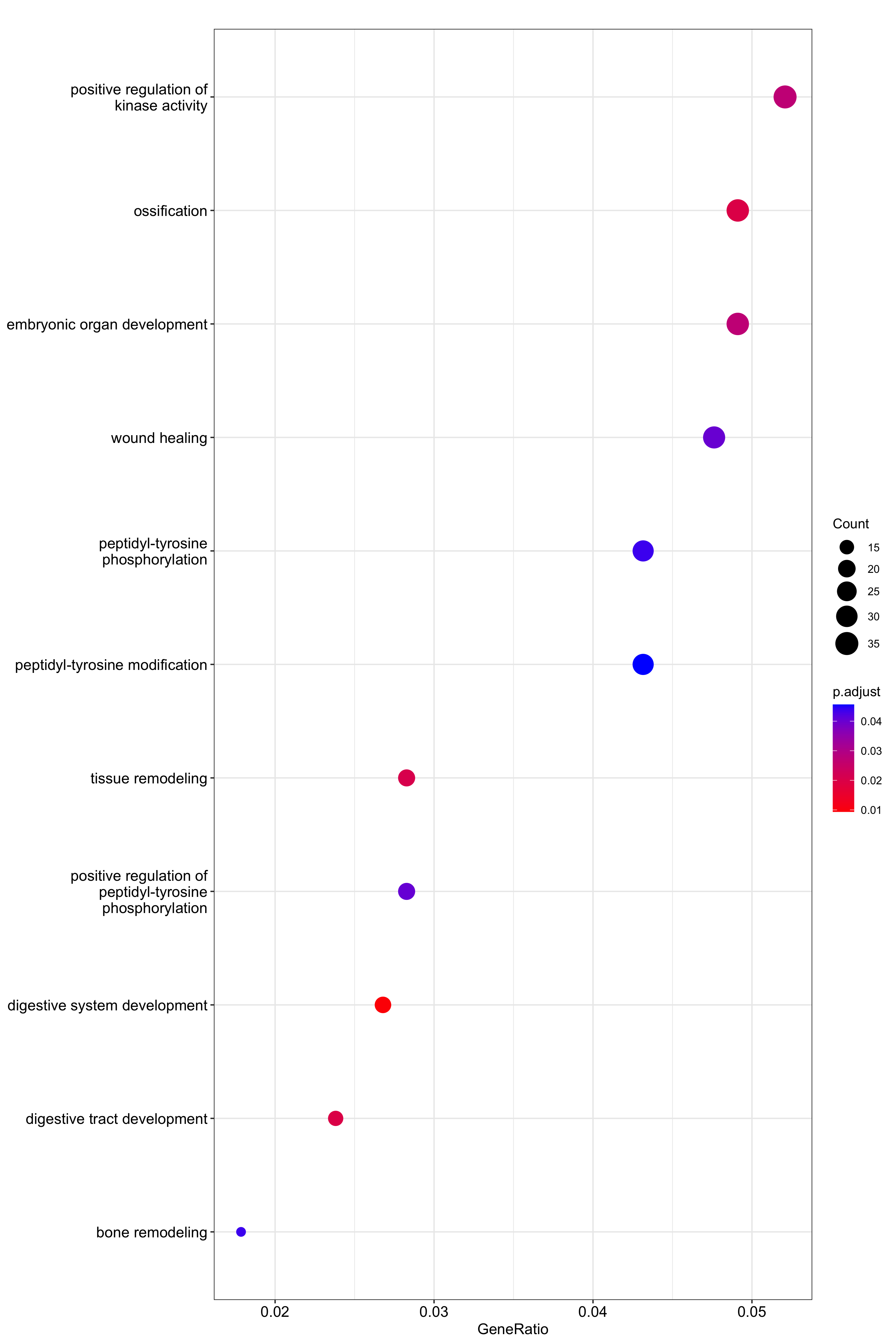

Supplement: Supplementary file 8 [file Image3.PNG]
